# Medical Opinion and Movement

**Published:** 1907-12-07

**Authors:** 


### Medical Opinion and Movement.

The winter session of the General Medical Council commenced on Tuesday, November 26, and was completed on the following day. In the opening address, the President, Dr. Donald Macalister, commended the efforts of the University of Wales to establish a medical faculty, and spoke approvingly of the high standard of professional attainment which was evidently intended. In regard to finance, he pointed out that recent endeavours to improve the efficiency of those admitted to the register tended to diminish the number of persons qualified, and therefore to reduce the available sources of the Council. In his opinion, therefore, the Council should hesitate before making fresh advances in this direction. Reference was made to the Bills in Parliament for the restriction of medical and dental practice by limited companies, and explanations were given for the failure to secure their passage. The President also alluded to the now famous case of Clifford *v.* Timms, which has been finally disposed of in the House of Lords, and expressed the view that in the light of this and other cases the law as it stands is capable, if fully enforced, of putting down some of the abuses which have been thought by many to require new legislation. Finally the Council were informed of certain steps that have already been taken in the provinces of Quebec and Nova Scotia by which reciprocal recognition of the medical qualifications granted in the Mother Country and in these provinces of the Dominion of Canada may be established.

The work of the Council consisted chiefly in inquiries into cases of alleged misconduct, but an important discussion took place upon the question of securing legislation to prohibit the practice of medicine and surgery by unqualified persons in this country. It was urged by Dr. Langley Browne that almost every other country, and also the Colonies, had in recent legislation taken power to secure this prohibition. There was a general consensus of opinion that further powers were urgently required to deal with the existing abuses, not only in the interest of registered medical practitioners, who are inadequately protected in their rights under existing conditions, but also in the interests of the public, who suffer largely through ignorance at the hands of charlatans and quacks. Finally, it was resolved to appoint a committee to ascertain what legal provisions exist in the Colonies and Dependencies of the Empire, and also in foreign countries, for the prevention of medical practice by other than legally qualified persons, and to consider what steps should be taken to procure effective legislation for the same purpose in this country. Subsequently a committee of eight members was formed for the purpose. The President, while recognising the value of the information which it was proposed to obtain, was not at all sanguine of the passage of such a Bill through the Houses of Parliament.

It is satisfactory to learn that the question at issue between the India Office and Mr. Haffkine has at last been settled. It will be remembered that in what is known as the Mulkowal disaster fatal results followed the injection of a certain antiplague serum prepared at the laboratory under the supervision of Mr. Haffkine. Although there seemed ample reason for supposing that contamination took place after the serum left the laboratory, the official inquiry which took place resulted in localising the probable source of contamination in the laboratory itself, and thereby laying the responsibility for the disaster at the very doors of Mr. Haffkine. Much indignation has been expressed at the decision, not only by Mr. Haffkine himself in self-defence, but also by a large number of his colleagues and scientific experts. Mr. Haffkine has now received a letter from the India Office saying that the Secretary of State recognises that an important body of scientific opinion is favourable to him in the question of the origin of the disaster, and that the Secretary's own attitude is indicated by the offer of employment upon honourable terms. Mr. Haffkine has replied, expressing gratitude for the expressions contained in it, accepting the offer, and stating his intention of proceeding to India at the earliest date. Apart from other considerations, it is satisfactory that in this way the services of so eminent a worker in the field of bacteriological research have been retained for the Indian Government.

There is perhaps no more troublesome and wearisome complaint for the general practitioner to treat than chronic suppurative otitis media. Short of operation, the treatment always resolves itself into the application—syringing or insufflation—of some antiseptic lotion or powder. Many are the applications used, and they vary with the seasons and the particular aurist consulted. For there is a fashion in these, as in other things: their determination largely depends upon individual experience, and is empirical rather than deductive. Any treatment, therefore, which is based upon some definite principle should be welcomed, and at least afforded a fair trial. Dr. A. F. Blagdon Richards, of the Swansea General Hospital, makes the reasonable suggestion that an antiseptic lotion, in order to permeate not only a minute perforation of the membrana tympani, but also the intricate recesses of the middle ear, should have a higher specific gravity than the pus which it is intended to displace. He has therefore adopted as a working formula the following lotion: Boric acid 1 drachm, rectified spirit of wine 2 or 3 drachms, and glycerine to make up to 1 ounce. The specific gravity of this lotion is about 1200, while pus is only about 1030. He advises that the ear be first syringed with boric acid lotion and carefully dried, and then the glycerin lotion freely dropped in. By this treatment he claims to have obtained considerable success in a large number of otherwise intractable cases, and he has published details of a series of typical cases treated in this way.

